# Quantitative Trait Locus Mapping of Salt Tolerance in Wild Rice *Oryza longistaminata*

**DOI:** 10.3390/ijms23042379

**Published:** 2022-02-21

**Authors:** Lei Yuan, Licheng Zhang, Xiao Wei, Ruihua Wang, Nannan Li, Gaili Chen, Fengfeng Fan, Shaoying Huang, Jianxiong Li, Shaoqing Li

**Affiliations:** 1 State Key Laboratory of Hybrid Rice, Hongshan Laboratory of Hubei Province, Key Laboratory for Research and Utilization of Heterosis in Indica Rice of Ministry of Agriculture, Engineering Research Center for Plant Biotechnology and Germplasm Utilization of Ministry of Education, College of Life Science, Wuhan University, Wuhan 430072, China; yuanlei0316@whu.edu.cn (L.Y.); 2020102040048@whu.edu.cn (L.Z.); 2020202040074@whu.edu.cn (X.W.); 2020202040082@whu.edu.cn (R.W.); 2020202040193@whu.edu.cn (N.L.); 2019202040073@whu.edu.cn (G.C.); fanfengfeng0603@163.com (F.F.); shaoyinghuang@whu.edu.cn (S.H.); 2 Hubei Provincial Key Laboratory for Protection and Application of Special Plants in Wuling Area of China, College of Life Sciences, South-Central University for Nationalities, Wuhan 430074, China; 3 State Key Laboratory for Conservation and Utilization of Subtropical Agro-Bioresour, College of Agriculture, Guangxi University, Nanning 530004, China; jxli@scbg.ac.cn

**Keywords:** QTLs, wild rice, *Oryza longistaminata*, salt tolerance, backcross inbred line

## Abstract

Salt stress is one of the most severe adverse environments in rice production; increasing salinization is seriously endangering rice production around the world. In this study, a rice backcross inbred line (BIL) population derived from the cross of 9311 and wild rice *Oryza longistaminata* was employed to identify the favorable genetic loci of *O. longistaminata* for salt tolerance. A total of 27 quantitative trait loci (QTLs) related to salt tolerance were identified in 140 rice BILs, and 17 QTLs formed seven QTL clusters on different chromosomes, of which 18 QTLs were derived from *O. longistaminata*, and a QTL for salt injury score (SIS), water content of seedlings (WCS) under salt treatment, and relative water content of seedlings (RWCS) was repeatedly detected and colocalized at the same site on chromosome 2, and a cytochrome P450 86B1 (MH02t0466900) was suggested as the potential candidate gene responsible for the salt tolerance based on sequence and expression analysis. These findings laid the foundation for further improving rice salt tolerance through molecular breeding in the future.

## 1. Introduction

Soil salinity is one of the major environmental stress factors limiting the growth and yield of rice around the world [1], more than one third of the world’s irrigated lands are salinized to varying degrees [2]. Rice, which feeds more than half of the world’s population, is the most salt-sensitive cereal crop plant [3]. Salt stress not only causes osmotic stress and ionic toxicity in plants [4], but also induces oxidative stress [5], leading to leaf damage and yield reduction, or even death. Developing salt-tolerant rice varieties is the most effective and economical way to improve yield and expand rice production in salinized areas. Usually, rice responds differently to salt stress at different growth stages; seedling and reproductive stages are relatively more sensitive, and germination, tillering, and maturity stages are relatively more tolerant to salt stress [6].

Salt tolerance is a comprehensive trait, and various physiological and biochemical responses to salt stress are controlled by a variety of genes, which has a complex genetic basis. Identification of salt tolerance loci in rice, especially in the seedling stage, will help to promote the development of salt-tolerant rice, just as exemplified by the RIL FL478 and Saltol derived from IR29 and Pokkali [7]. So far, a large number of salt-tolerant associated QTLs have been identified using different populations, such as recombinant inbred lines (RILs) [8,9,10,11], and a few influential salt-tolerant loci, such as *SKC1*, have been fine mapped or cloned using map-based cloning strategies [10,12]. However, less QTLs related to salt tolerance have been reported from wild rice *Oryza longistaminata*.

Wild rice is an important gene pool for rice yield, quality, and resistance improvement [13,14]. *O. longistaminata*, an African wild rice species variety of AA genome, has a good performance in abiotic tolerance, but only a few genes have been discovered and there is little research on rice breeding [15,16,17,18]. In order to further explore the alien genes related to salt tolerance and broaden the genetic diversity of rice, 140 backcross inbred lines (BILs) derived from the cross of 9311 and wild rice *O. longistaminata* were investigated, and the salt injury indexes were scored based on evaluation of the characteristics, including seedling length, root length, seedling weight, and root weight. A total of 27 QTLs for seven salt-tolerant traits were identified in the BIL population in two separate experiments, of which 18 QTLs were derived from *O. longistaminata*, and 17 loci formed seven QTL clusters on different chromosomes. These findings will effectively broaden the genetic resources and help to improve the salt tolerance of rice.

## 2. Results

### 2.1. Phenotype Observation of the BIL Population under Salt Treatment

In order to know if the wild rice *O. longistaminata* harbor favorable genes for salt tolerance, we investigated the performance of the *O. longistaminata* BIL lines treated with 150 mM NaCl solution for seven days at seedling stage, using the parent 9311 as a control. Results showed that the BILs exhibited significant differences for salt tolerance; the salt injury scores ranged among 3–9 (Table 1) and showed very large coefficient of variation in BILs, indicating that the BILs had rich genetic diversity (Table S1). In the first test, the seedling length of the control ranged from 17.31 to 54.62 cm, with an average of 30.61 cm, while the seedling length of the treatment ranged from 13.57 to 34.71 cm, with an average of 21.40 cm. The average root length was 8.38 cm for the control and 8.02 cm for the salt treatment. In the second test, the average seedling length was 28.70 cm and 22.46 cm for the control and salt treatment, respectively. The average root length was 7.05 cm for the control and 6.32 cm for the salt treatment. The root length was apparently less damaged relative to the shoots in the two tests, reflecting that the shoot is more sensitive to salt stress than the root.

In the first test, the average shoot fresh weight was 229.62 mg and 62.10mg for the control and salt treatment, respectively. The average shoot dry weight was 33.58 mg for the control and 22.00 mg for the salt treatment, and the average root dry weight was 4.71 mg for the control and 3.36 mg for treatment (Table S1). In the second test, the average shoot fresh weight was 196.75 mg and 45.72 mg for the control and salt treatment, respectively. The average shoot dry weight was 26.97 mg for the control and 18.49 mg for the salt treatment, and the average root dry weight was 4.00 mg and 3.07 mg for the control and salt treatment, respectively (Table S1). Among the three investigated traits, fresh weight was the most vulnerable, implying that maintaining high content of water in rice tissues may play a central role in enhancing the resistance of rice against salt stress. 

**Figure 1:**
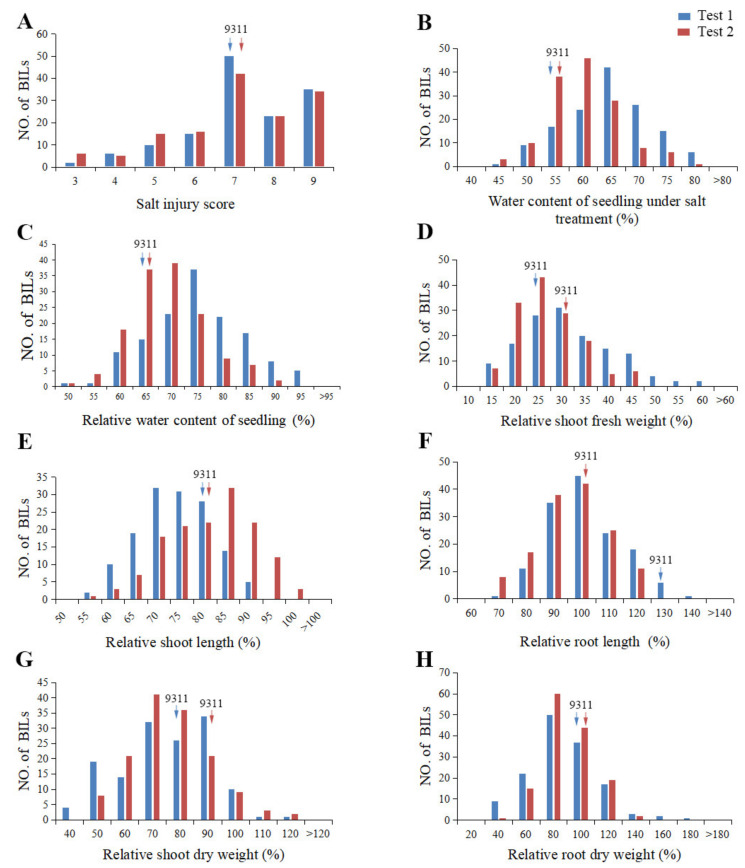
Frequency distribution of eight traits related to salinity tolerance of the 140 BILs seedlings. (**A**), the *X*-axis represents the salt injury score; (**B**–**H**), the *X*-axis represents the relative value of different traits, respectively. (**A**–**H**), the *Y*-axis shows the number of BILs. Arrows indicate the phenotypes of 9311.

Further analysis showed that the salt-related trait of the BILs exhibited a normal or skewed distribution pattern (Figure 1). All the investigated characters showed a similar trend in the two experiments, although the salt injury score (SIS), water content of seedling under salt treatment (WCSST), relative water content of seedling (RWCS), relative root length (RRL), and relative shoot fresh weight (RSFW) in the first test were slightly higher than the second, while the relative shoot length (RSL), relative shoot dry weight (RSDW), and relative root dry weight (RRDW) were slightly lower, suggesting that the data are consistent in the two tests, which could be well used for further QTL analysis (Table 1).

### 2.2. Correlation of the Salt-Tolerance-Related Traits

**Figure 2:**
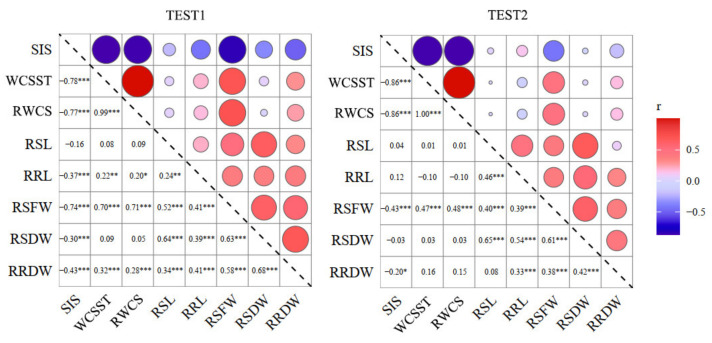
Correlation analysis of salt-tolerant traits of BILs. Notes: SIS, salt injury score; WCSST, water content of seedling under salt treatment; RWCS, relative water content of seedling; RSL, relative shoot length; RRL, relative root length; RSFW, relative shoot fresh weight; RSDW, relative shoot dry weight; RRDW, relative root dry weight. *, ** and *** means significance at the 0.05, 0.01 and 0.001 level, respectively (2-tailed); r, correlation.

In order to further understand the potential relationship of the salt-tolerant traits, the correlation of eight traits of 140 BILs was analyzed. Results showed that most of the traits were significantly correlated; the RSFW showed a significant correlation with all the other traits in the two tests. SIS was negatively correlated with all other traits except for RSL and RRL, indicating that, as salt injury increases, shoot and root growth are stunted, which ultimately leads to seedling death (Figure 2). The correlation between SIS and WCSST was extremely significant in the two tests, with a maximum of −0.863, which translates to higher water content rate of plants with the least injury. Meanwhile, WCSST and RWCS were almost 100% correlated to each other (Figure 2), meaning that any of them can replace each other in scoring the salt tolerance of rice seedlings.

### 2.3. QTL Mapping of the Salt Tolerance of the O. longistaminata BIL Population

**Figure 3:**
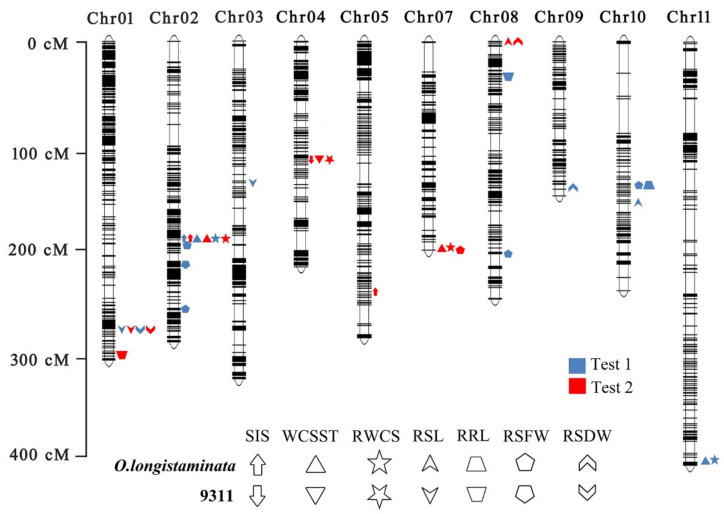
QTLs identified in the *O. longistaminata* for salt tolerance. Left is the scale for the genetic length of each chromosome. The upward direction indicates the locus comes from *O. longistaminata*, while the downward direction indicates the locus comes from 9311. The blue indicates QTLs detected in the first experiment and the red indicates QTLs detected in the second experiment. cM, centi-Morgan.

In total, 27 QTLs were identified for seven physiological and morphological traits from the *O. longistaminata* BIL population at the seedling stage, which were located on all chromosomes except chromosome 6 and 12 (Figure 3 and Table S2). Among them, 18 QTLs were derived from wild rice *O. longistaminata*; two QTLs for the salt injury score, named as *qSIS2* and *qSIS5*, were mapped on chromosome 2 and 5 and explained 20.5% and 6.9% of the phenotypic variations, respectively (Table S2). Three QTLs for water content of seedling, *qWCSST2*, *qWCSST7*, and *qWCSST11*, which were mapped on chromosomes 2, 7, and 11, explained 20.4%, 5.0%, and 8.5% of the phenotypic variations, respectively. There were three QTLs for relative water content of seedling, named as *qRWCS2*, *qRWCS7*, and *qRWCS11*, that explained phenotypic variations of 22.3%, 4.9%, and 8.3%, respectively. Two QTLs, named as *qRSL8* and *qRSL10*, were detected for relative shoot length and explained the phenotypic variations of 6.4% and 10.0%, respectively. Only one QTL was found for relative root length, *qRRL10*, which explained 8.3% of the phenotypic variations. There were five QTLs, *qRSFW2.1*, *qRSFW2.3*, *qRSFW7*, *qRSFW8*, and *qRSFW10*, detected for the relative shoot fresh weight, explaining 8.3%, 10.1%, 10.7%, 9.6%, and 13.6% of the phenotypic variations, respectively. Two QTLs, *qRSDW8* and *qRSDW9*, responsible for relative shoot dry weight explained phenotypic variations of 7.8% and 6.9%, respectively. Of these, the *qSIS2*, *qWCSST2*, and *qRWCS2* from *O. longistaminata* flanked by Bin 2-116 and Bin 2-117 on chromosome 2 were repeatedly identified in the two tests, the *qRRL10* and *qRSFW10* were found overlapped and flanked by Bin 10-10 and Bin 10-11, and both the *qRSL8* and *qRSDW8* were also mapped in one locus, indicating that these loci from *O. longistaminata* have pleiotropic effects, and show potentially important value in rice breeding practice.

Additionally, there were nine QTLs, including *qRSL1*, *qRSDW1*, *qSIS4*, *qWCSST4*, *qRWCS4, qRSL3*, *qRSFW2.2*, *qRRL1*, and *qRRL8*, for salt tolerance identified from 9311 (Figure 3 and Table S2), of which *qSIS4*, *qWCSST4*, and *qRWCS4*, explaining the phenotypic variations ranging from 6.9% to 11.2%, were overlapped on chromosome 4. The *qRSL1* and *qRSDW1* were detected in the two tests, suggesting that this locus may also have strong functions in salt tolerance.

### 2.4. Confirmation of the QTLs for Salt Tolerance

**Figure 4:**
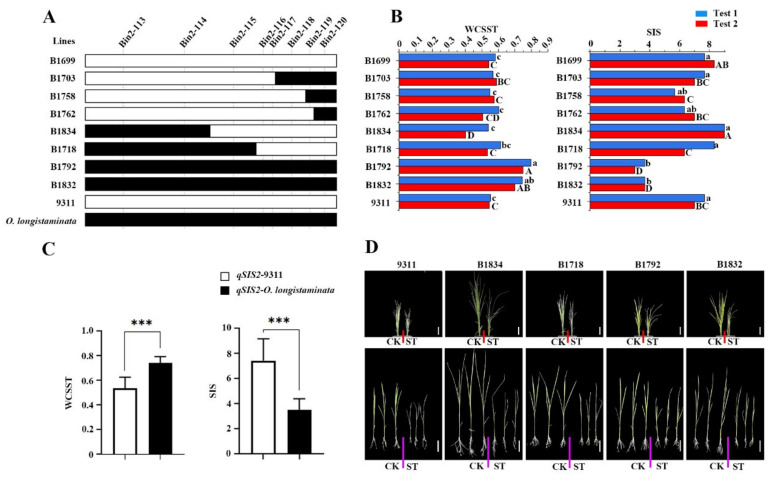
Validation of the function of *qWCSST2/qSIS2/qRWCS2*. (**A**) Genotype of the representative BIL lines. (**B**) Phenotypic value corresponding to BILs. (**C**) Average of the WCSST and SIS value of the representative BIL lines with and without *qWCSST2/qSIS2/qRWCS2*. The white rectangle shows the homozygous genotype from parent 9311, including BIL 1699, 1703, 1758, B1762, 1834, 1718. The black rectangle indicates the heterozygote genotype, and contains BIL 1792 and 1832. *** *p* < 0.001. (**D**) The phenotype of the representative BILs and the parent 9311, bar = 5 cm. The red and purple lines separate control and salt-treated seedlings, with the control seedlings on the left and the salt-treated seedlings on the right.

To further verify the function of the novel QTLs for salt tolerance from *O. longistamianta*, we compared the genotypes and genetic effects between the representative BIL lines with and without *qSIS2/qWCSST2/qRWCS2*. Results showed that the Bin 2-116 and Bin 2-117 well determined the core interval of *qSIS2/qWCSST2/qRWCS2* in the BIL lines (Figure 4A); the BIL line 1792 and 1832, which harbor *qSIS2/qWCSST2/qRWCS2*, showed significantly higher water content of shoot under stress treatment and less injury scores than the BIL lines, such as BIL1699, 1703, 1758, 1762, 1834, and 1718, without the corresponding QTLs (Figure 4B). Meanwhile, the average water content under treatment of the BIL 1792 and 1832 were about 21% higher than that of the lines 1699, 1703, 1758, 1762, 1834, and 1718, and the SIS of the lines 1792 and 1807 was also significantly lower than that of BIL1699, 1703, 1758, 1762, 1834, and 1718 (Figure 4C), indicating that the *qSIS2/qWCSST2/qRWCS2* from *O. longistaminata* could significantly improve the salt tolerance of rice (Figure 4D).

**Figure 5:**
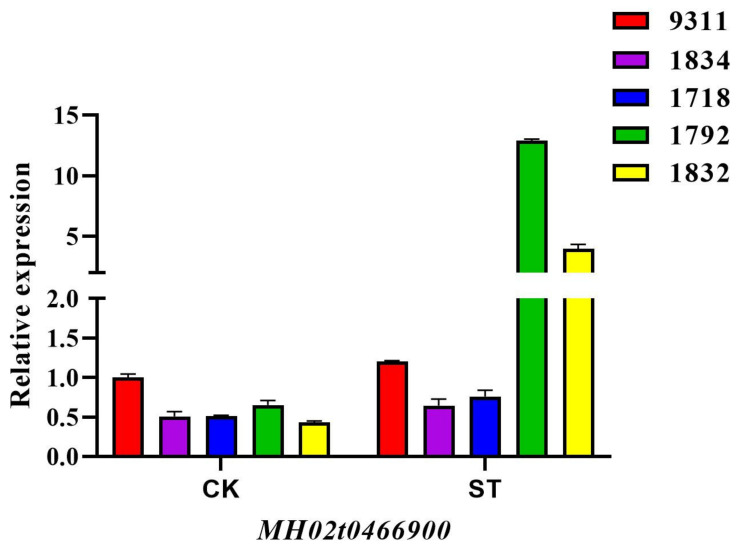
qPCR analysis of the expression of candidate gene *MH02t0466900* in seedlings under salt stress. The BIL lines of B1792 and B1832 contained *qSIS2/qWCSST2/qRWCS2* locus, while the 1834 and 1718 did not contain the QTL.

Genomic structural analysis showed that *qSIS2/qWCSST2/qRWCS2* delimited by Bin 2-116 and Bin 2-117 was about 89 Kb referenced to the MH63 genome (https://rice.hzau.edu.cn/rice_rs1/ (accessed on 17 September 2021)), and a total of 15 candidate genes, including 2 homologous genes, 3 hypothetical proteins, 1 expressed protein, and 10 annotated genes, were found within the region (Table 2). qRT-PCR analysis showed that the *MH02t0466900* were highly induced in the tolerant BIL seedlings after 4 days of salt treatment (Figure 5), while no change or a slight decrease was observed in the parent 9311 and the BILs without the corresponding QTL (Figure S1). The *MH02t0466900*, which encodes a cytochrome P450 86B1, increased over at least 15 folds relative to the untreated controls in the BIL lines with *qSIS2/qWCSST2/qRWCS2*. The *MH02t0466900* gene sequence does not contain introns, and its coding sequence is consistent with the genome sequence. Sequence analysis showed that the coding region of *MH02t0466900* had great number of variations between 9311 and *O. longistaminata* (Figure S2), which lead to seven amino acid mutations and eight more amino acids in the posterior segment of the cytochrome P450 86B1 in 9311 (Figure S3), and further great difference of the protein structure between 9311 and *O. longistaminata* (Figure S4). These differences may result in the functional variation of *MH02t0466900* between 9311 and *O. longistaminata*, and, hence, the increase in salt tolerance of BIL lines with *qSIS2/qWCSST2/qRWCS2*.

## 3. Discussion

At present, there are many indicators suggested to evaluate salt tolerance; the most direct method is to measure the content of Na^+^, K^+^, and Na^+^/K^+^ ratio. Other indicators commonly used include salt injury score, shoot length, root length, relative shoot length, relative root length, dry weight and relative dry weight, and biomass [19,20]. Here, we found that the water content is the most sensitive to the salt treatment than the others (Table 1); correlation analysis revealed that the water content not only showed the highest coefficient of variation with the salt injure score, but also showed a relatively stable and high correlation with the other five indicators (Figure 2), meaning that the water content in rice tissues is easily disturbed by the salt stress. Importantly, the *qSIS2* for salt injury score and the *qWCSST2* for water content were overlapped in our research (Figure 4), indicating that the water content can well reflect the injury status of rice under salt stress and could be used as a critical indicator for rice salt-tolerance evaluation [21]. 

In previous studies, a great number of QTLs associated with salt tolerance have been identified, and most of them are discovered in cultivated rice [22,23,24,25], although a few QTLs related to salt injury are found in wild rice. Tian et al. (2011) identified 13 QTLs for salt tolerance using a set of introgression lines derived from common wild rice *O. rufipogon*, which could improve salt tolerance in the background of cultivated rice Teqiing, and the *qSTS2* for salt tolerance score explained 9% of the phenotypic variance that was mapped on chromosome 2 [26]. Similarly, another QTL for salt tolerance score, named as *qSIS2*, which explained 6.6% of the phenotypic variance, was also detected on chromosome 2 in wild rice by Amoah et al. [27]; both of the QTLs for salt tolerance were very close to the *qSIS2.1* detected in our study (Figure 3, Table 2), implying that this locus may be a critical region for clustering of the important genes against salt stress in rice. 

At present, many salt tolerance genes have been reported in plants, and also found in rice. Plant responses to salt stress are pleiotropic, occurring at the organismic, cellular, and molecular levels. These genes have the functions of ion homeostasis, osmotic regulation, scavenging of reactive oxygen species, and nutrient balance, and also regulate various traits of plants under salt stress [28]. With overexpressed rice Na^+^/H^+^ exchangers (*OsNHX1*) and H^+^-pyrophosphatase in tonoplasts (*OsVP1*) in a japonica elite rice cultivar, Zhonghua 11, the transgenic plants had stronger salt tolerance and higher survival rate. The seedling height, root length, and fresh weight of the transformed lines were also significantly increased, and the concentration of Na^+^ in leaves was lower than WT [29]. The *OsDRAP1* gene, which encodes an ERF transcription factor, when overexpressed, improved salt tolerance and increased the survival rate of rice seedling under salt stress [30].

In this study, the *qSIS2/qWCSST2/qRWCS2* was repeatedly detected in the BIL population, indicating that this locus has pleiotropic and stable genetic effects on rice salt tolerance. Expression analysis showed that *MH02t0466900*, which encodes a cytochrome P450 86B1, was highly induced by salt stress (Figure 5). Correspondingly, the amino acid and protein structure encoded by *MH02t0466900* exhibit great variations between the wild rice and 9311 (Figure S3 and S4). Interestingly, the cytochrome P450 CYP86A1 encodes a fatty acid omega-hydroxylase involved in suberin monomer biosynthesis in Arabidopsis [31]. Suberin deposition affects root epidermal formation, regulates Na^+^ penetration, and affects plant salt tolerance by affecting ion homeostasis. The expression of OS03T0140200-01 (cytochrome P450 86B1-like) gene in rice was upregulated in salt stress [32]; recent reports have reported that heterologous expression of cytochrome P450 94B1 increased the salt tolerance and root suberin deposition in Arabidopsis and rice (*Oryza sativa*) seedlings [33]. These findings mean that *MH02t0466900* from *O. longistaminata* is most possibly responsible for the increase in salt tolerance in rice; we will verify this result in future experiments using transgenic plants. Wild rice backcross inbred line has been suggested as a powerful tool for rice improvement; the QTLs found in wild rice *O. longistaminata* in our study can be directly transferred to cultivated rice by crossing the BIL lines, and it will greatly enrich the genetic basis if we employed them in rice breeding practice. 

## 4. Materials and Methods

### 4.1. Plant Materials and Salt Treatments

A set of 140 BC_2_F_20_ BIL lines derived from *O. longistaminata* and acceptor parent 9311 were used in the experiment [16,17,18]. The seeds of the 9311 and BILs were placed in an oven at 50 °C for 5 days to break the possible dormancy, then germinated at room temperature for 48 h after surface sterilization with 2% sodium hypochlorite solution for 10 min and rinsing well with distilled water. The 96-well (12 × 8) PCR plates with removed well bottoms were used for sowing, and a total of 8 seeds were sown in one row of each line as a technical replicate. These plates were transferred into Yoshida’s nutrient solution [34] after floating on water for 3 days. The culture solution was refreshed every three days and the pH was maintained at 5.4. The seedlings were grown in a 12-h light/12-h dark photoperiod at 28 °C. Each line was planted with three technical replications according to completely randomized designs; the average of individual replicates was treated as the phenotypic value. The whole experiment was biologically replicated twice.

### 4.2. Phenotype Scores and Correlation Analysis

After sowing of 15 days, the seedlings were divided into two sets. One set of seedlings was transferred to cultural solution with 150 mM NaCl, and the other set of seedlings was kept in Yoshida’s solution as a control [34]. After 7 days of treatment, the backcross inbred lines were scored for visual salt injury score (SIS) based on a modified standard evaluation system at seedling stage [35]. The score is based on the following: score 1—normal growth, no leaf symptoms; score 3—nearly normal growth, only the tips of few leaves whitish and rolled; score 5—growth severely retarded, most leaves rolled, only a few are elongating; score 7—complete cessation of growth, most leaves dry, some plants dying; score 9—almost all plants dead or dying. The seedling length (SL), root length (RL), and shoot fresh weight (SFW) of each line of salt treatment and control group were subsequently measured. The shoots and roots were then rinsed with distilled water several times, packed in craft paper bags, respectively, and dried at 80 °C for 5 days. Then, the shoot dry weight (SDW) and root dry weight (RDW) of the control group and salt treatment were measured separately. 

Water content of seedlings (WCS) was calculated according to the following formula: WCS (%) = (SFW − SDW)/SFW × 100. Relative water content of seedlings (RWCS), relative shoot length (RSL), relative root length (RRL), relative shoot fresh weight (RSFW), relative shoot dry weight (RSDW), and relative root dry weight (RRDW) were calculated according to the following formula: relative value (%) = (trait value under salt stress)/(trait value under control) × 100.

The statistical analyses were performed with SPSS Statistics 20 (IBM, Armonk, NY, U.S.A.). Correlation analysis was calculated using R-studio. The R Corrplot package was installed and loeaded to analyze the correlation coefficients between the two variables. Correlation among traits was computed at *p* < 0.05 and *p* < 0.01, respectively.

### 4.3. QTL Mapping

A total of 2432 bin markers were used to construct the genetic linkage map covering the whole-genome sequencing (unpublished data). The DNA extraction, single nucleotide polymorphism (SNP) calling, genotyping, bin map, and genetic linkage map construction were all performed following previous reports [16,17,18]. The QTL IciMapping v4.1 software (Chinese Academy of Agricultural Sciences, Beijing, China) was used to construct the linkage map and QTL analysis [36], and the limit of detection (LOD) threshold was set at 2.5.

### 4.4. Real-Time Quantitative PCR

The total RNA from the shoots of rice was extracted using the RNAprep Pure Plant Kit (Tiangen, Beijing, China). The cDNA for real-time PCR was reverse-transcribed from 2 μg of total RNA using the Hifair^®^ II 1st Strand cDNA Synthesis Kit (gDNA digester plus) (Yeasen, Shanghai, China), and the real-time PCR was carried out using a Hieff^®^ qPCR SYBR Green Master Mix (No Rox) (Yeasen, Shanghai, China) in Real-Time PCR machine (Bio-Rad, Hercules, CA, USA) according to the manufacturer’s instructions. The rice *Ubi* was selected as the internal control. Primers used for qRT-PCR analysis are listed in Table S3.

### 4.5. Gene Sequence Comparison and Protein Structure Prediction

Genomic DNA of 9311 and *O. longistaminata* was extracted by CTAB method. Primers were designed in 3′ UTR, 5′ UTR regions, and the middle of the gene sequences, and the gene sequences were divided into several segments for PCR amplification, which was about 1kb in length. Primers used for PCR amplification are listed in Table S4. After sequencing, the software DNAMAN was used to compare the assembled genome sequence between 9311 and *O. longistaminata*, and the amino acid sequence was predicted for comparison. Protein structure prediction is performed on the website (https://swissmodel.expasy.org/ (accessed on 24 January 2022)).

## Figures and Tables

**Table 1:** The main performance of *O. longistaminata* BILs under salt treatment at seedling stage.

Tests	Items	9311	BILs		
			Mean ± SD	CV (%)	Range
Test 1	SIS	7.00	6.83 ± 1.45	21.24	3.00–9.00
	WCSST (%)	55.22	62.09 ± 7.63	12.29	42.82–79.69
	RWCS (%)	64.40	72.78 ± 8.99	12.35	49.95–94.60
	RSL (%)	79.13	70.88 ± 7.64	10.78	53.25–89.91
	RRL (%)	120.71	96.43 ± 12.5	12.97	63.28–135.45
	RSFW (%)	25.35	28.89 ± 9.76	33.79	12.14–56.19
	RSDW (%)	78.90	70.06 ± 16.15	23.06	35.16–114.39
	RRDW (%)	81.73	77.28 ± 25.15	32.54	26.85–177.20
Test 2	SIS	7.00	6.71 ± 1.63	24.22	3.00–9.00
	WCSST (%)	54.50	57.65 ± 6.52	11.30	40.23–77.33
	RWCS (%)	64.39	66.98 ± 7.51	11.21	47.32–89.48
	RSL (%)	79.15	78.63 ± 9.76	12.41	52.35–97.03
	RRL (%)	95.29	91.24 ± 12.46	13.66	61.72–117.00
	RSFW (%)	27.20	24.51 ± 6.98	28.46	12.30–44.70
	RSDW (%)	81.43	71.00 ± 13.95	19.65	42.04–114.39
	RRDW (%)	88.37	80.44 ± 18.77	23.33	35.76–139.68

Note: SIS, salt injury score; WCSST, water content of seedlings under salt treatment; RWCS, relative water content of seedling; RSL, relative shoot length; RRL, relative root length; RSFW, relative shoot fresh weight; RSDW, relative shoot dry weight; RRDW, relative root dry weight; SD, standard deviation; CV, coefficient of variation.

**Table 2:** The predicted functional genes at the loci of *qSIS2/qWCSST2/qRWCS2*.

Loci	Gene Names	Functional Annotation
*qSIS2*	*MH02t0465500*	NAC domain-containing protein 8
	*MH02t0465600,* *MH02g0465700*	Beta-1,4-mannosyl-glycoprotein, 4-beta-*N*-acetylglucosaminyltransferase
	*MH02t0465800*	hypothetical protein OsI_07895
	*MH02t0465900*	hypothetical protein OsI_07896
	*MH02t0466000*	unnamed protein product
	*MH02g0466100*	Isocitrate dehydrogenase (NAD) regulatory subunit 1, mitochondrial
	*MH02g0466200*	Hypothetical protein
	*MH02g0466300*	Elongation factor Tu, chloroplastic
	*MH02t0466400*	Putative eukaryotic translation initiation factor
	*MH02t0466500*	High-affinity nitrate transporter 3.1 Partner protein for high-affinity nitrate transport OsNAR2.1
	*MH02t0466600*	Rhodanese-like domain-containing protein 11, chloroplastic
	*MH02t0466700*	Nucleoporin GLE1
	*MH02t0466800*	Endoglucanase E1
	*MH02t0466900*	Cytochrome P450 86B1

## Data Availability

The data presented in this study are available in the article.

## Supplementary Materials

The following supporting information can be downloaded at: https://www.mdpi.com/article/10.3390/ijms23042379/s1.

## Abbreviations

SIS	salt injury score
WCSST	water content of seedlings under salt treatment
RWCS	relative water content of seedling
RSL	relative shoot length
RRL	relative root length
RSFW	relative shoot fresh weight
RSDW	relative shoot dry weight
RRDW	relative root dry weight
SD	standard deviation
CV	coefficient of variation
