# Supplementary material for: Quantitative Trait Locus Mapping of Salt Tolerance in Wild Rice Oryza longistaminata
Source: Int J Mol Sci. 2022 Feb 21;23(4):2379. doi: 10.3390/ijms23042379 (PMC8878134; doi:10.3390/ijms23042379)
Supplement: Supplementary file 1 [file ijms-23-02379-s001.zip › ijms-1592653-supplementary.pdf]

Quantitative Trait Locus Mapping of Salt Tolerance in Wild Rice *Oryza longistaminata*

Lei Yuan[1, 2], Licheng Zhang[1], Xiao Wei[1], Ruihua Wang[1], Nannan Li[1], Gaili Chen[1], Fengfeng Fan[1], Shaoying Huang[1], Jianxiong Li[3], Shaoqing Li[1, *]

[1] State Key Laboratory of Hybrid Rice, Hongshan Laboratory of Hubei Province, Key Laboratory for Research and Utilization of Heterosis in Indica Rice of Ministry of Agriculture, Engineering Research Center for Plant Biotechnology and Germplasm Utilization of Ministry of Education, College of Life Science, Wuhan University, Wuhan 430072, China.

[2] Hubei Provincial Key Laboratory for Protection and Application of Special Plants in Wuling Area of China, College of Life Sciences, South-Central University for Nationalities, Wuhan, Hubei, P.R. China

[3] College of Agriculture, Guangxi University, Nanning 530004, China.

* Correspondence: Shaoqing Li, shaoqingli@whu.edu.cn

Table S1. Performance of *O. longistaminata* BILs under salt treatment at seedling stage

Tests	Traits	Items	9311	BILs		
				Mean ± SD	CV (%)	Range
Test 1	SIS	Treatment	7.00	6.83±1.45	21.24	3.00~9.00
	WCS (%)	Treatment	55.22	62.09±7.63	12.29	42.82~79.69
		CK	85.74	85.32±1.49	1.74	79.22~93.18
		RWCS (%)	64.40	72.78±8.99	12.35	49.95~94.60
	SL (cm)	Treatment	22.91	21.40±4.70	21.99	13.57~34.71
		CK	28.96	30.61±8.27	27.00	17.31~54.62
		RSL (%)	79.13	70.88±7.64	10.78	53.25~89.91
	RL (cm)	Treatment	12.74	8.02±1.55	19.28	3.77~11.73
		CK	10.55	8.38±1.62	19.35	3.55~12.36
		RRL (%)	120.71	96.43±12.5	12.97	63.28~135.45
	SFW (mg)	Treatment	60.90	62.10±19.90	32.04	28.19~134.25
		CK	240.19	229.62±83.90	36.54	107.11~564.63
		RSFW (%)	25.35	28.89±9.76	33.79	12.14~56.19
	SDW (mg)	Treatment	27.13	22.00±4.51	20.49	11.38~36.65
		CK	34.39	33.58±12.25	36.48	9.94~84.72
		RSDW (%)	78.90	70.06±16.15	23.06	35.16~114.39
	RDW (mg)	Treatment	3.88	3.36±0.94	27.85	1.37~6.74
		CK	4.75	4.71±1.75	37.21	1.32~10.92
		RRDW (%)	81.73	77.28±25.15	32.54	26.85~177.20
Test 2	SIS	Treatment	7.00	6.71±1.63	24.22	3.00~9.00
	WC (%)	Treatment	54.50	57.65±6.52	11.30	40.23~77.33
		CK	84.65	86.06±0.63	0.73	83.92~87.48
		RWCS (%)	64.39	66.98±7.51	11.21	47.32~89.48
	SL (cm)	Treatment	21.70	22.46±4.46	19.86	15.32~34.51
		CK	27.42	28.70±7.83	27.28	17.64~51.28
		RSL (%)	79.15	78.63±9.76	12.41	52.35~97.03

RL (cm)	Treatment	8.13	6.32±1.00	15.77	3.92~9.29
	CK	8.53	7.05±1.09	15.40	4.25~10.41
	RRL (%)	95.29	91.24±12.46	13.66	61.72~117.00
SFW (g)	Treatment	54.86	45.72±11.91	26.05	29.94~115.67
	CK	201.43	196.75±60.66	30.83	95.57~561.19
	RSFW (%)	27.20	24.51±6.98	28.46	12.30~44.70
SDW (g)	Treatment	25.17	18.49±2.94	15.89	12.56~27.23
	CK	30.90	26.97±7.16	26.53	9.94~51.08
	RSDW (%)	81.43	71.00±13.95	19.65	42.04~114.39
RDW (g)	Treatment	4.25	3.07±0.63	20.34	1.42~4.95
	CK	4.81	4.00±0.95	23.71	2.10~7.40
	RRDW (%)	88.37	80.44±18.77	23.33	35.76~139.68

Note: SIS, salt injury score; WCS, water content of seedling; SL, seedling length; RL, root length; SFW, shoot fresh weight; SDW, shoot dry weight; RWCS, relative water content of seedling; RDW, root dry weight. RSL, relative shoot length; RRL, relative root length; RSFW, relative shoot fresh weight; RSDW, relative shoot dry weight; RRDW, relative root dry weight; SD, standard deviation; CV, coefficient of variation.

Table S2. QTLs for salt tolerance based on the eight agronomic traits in the BIL population.

Traits	QTLs	Test	Chr.	L/Bin	R/Bin	L/bp	R/bp	LOD	Add	PVE(%)	Reported
SIS	*qSIS2*	Test1	2	2-116	2-117	22575088_22611985	22611986_22665065	3.9	-0.83	12.0	
		Test2	2	2-116	2-117	22575088_22611985	22611986_22665065	9.5	-1.33	20.5	
	qSIS4	Test2	4	4-122	4-123	15284013_15312179	15139044_15284012	3.4	2.75	6.9	
	qSIS5	Test2	5	5-46	5-47	23991146_24235101	23239592_23260495	3.5	-0.53	6.9	
WCSST	*qWCSST2*	Test1	2	2-116	2-117	22575088_22611985	22611986_22665065	4.3	0.04	11.6	
		Test2	2	2-116	2-117	22575088_22611985	22611986_22665065	10.5	0.06	20.4	
	qWCSST4	Test2	4	4-122	4-123	15284013_15312179	15139044_15284012	4.5	-0.11	11.2	
	qWCSST7	Test2	7	7-136	7-137	27823837_27938640	27764507_27823836	2.9	0.02	5.0	
	qWCSST11	Test1	11	11-101	11-102	29915279_29974136	29977198_30302103	3.1	0.03	8.5	
RWCS	*qRWCS2*	Test1	2	2-116	2-117	22575088_22611985	22611986_22665065	4.3	0.05	11.8	
		Test2	2	2-116	2-117	22575088_22611985	22611986_22665065	10.9	0.07	22.3	
	qRWCS4	Test2	4	4-122	4-123	15284013_15312179	15139044_15284012	4.4	-0.13	9.9	
	qRWCS7	Test2	7	7-136	7-137	27823837_27938640	27764507_27823836	2.8	0.02	4.9	
	qRWCS11	Test1	11	11-101	11-102	29915279_29974136	29977198_30302103	3.0	0.04	8.3	
RSL	*qRSL1*	Test1	1	1-161	1-162	36539238_36569505	36515725_36536296	5.0	-0.03	14.1	
		Test2	1	1-161	1-162	36539238_36569505	36515725_36536296	8.9	-0.05	24.7	
	qRSL3	Test1	3	3-170	3-171	14600244_14615147	14615527_14633578	2.6	-0.02	7.1	
	qRSL8	Test2	8	8-129	8-130	669244_742639	607302_665293	2.6	0.03	6.4	
	qRSL10	Test1	10	10-136	10-137	15029044_15130486	14519180_15029043	3.6	0.04	10.0	

	qRRL1	Test2	1	1-136	1-137	39449569_39525930	39173818_39449568	8.0	-0.08	23.4
RRL	*qRRL8*	Test1	8	8-96	8-97	3789343_3953980	3544197_3789193	2.8	-0.05	7.0
	qRRL10	Test1	10	10-10	10-11	12626156_12698496	12698497_13747806	3.2	0.04	8.3
	qRSFW2.1	Test1	2	2-51	2-52	30194849_30262012	29773088_29808296	4.4	0.03	8.3
	qRSFW2.2	Test2	2	2-92	2-93	25322209_25564628	25240940_25322208	4.5	-0.02	7.4
RSFW	*qRSFW2.3*	Test2	2	2-114	2-115	22903285_23106237	22665066_22903284	6.0	0.04	10.1
	qRSFW7	Test2	7	7-134	7-135	27938641_28103812	28103813_28549563	6.4	0.03	10.7
	qRSFW8	Test1	8	8-153	8-154	21471995_21575481	21453964_21471994	5.2	0.07	9.6
	qRSFW10	Test1	10	10-10	10-11	12626156_12698496	12698497_13747806	6.8	0.05	13.6
	qRSDW1	Test2	1	1-159	1-160	36587534_36864307	36569506_36587533	8.4	-0.07	22.1
		Test1	1	1-159	1-160	36587534_36864307	36569506_36587533	13.2	-0.10	29.2
RSDW	*qRSDW8*	Test2	8	8-129	8-130	669244_742639	607302_665293	3.2	0.04	7.8
	qRSDW9	Test1	9	9-10	9-11	20585490_20741414	20288114_20585489	3.6	0.10	6.9

Table S3. Primers used for qRT-PCR analysis.

Genes	F	R
MH02t0465500	CTACCAGTTGCATGGAAAAC	TGGGATCGTAATTTTCTGTT
MH02t0465600	GCGGGCTACTACAACTGCAA	CACTTCAACCTTGACATGCT
MH02t0466000	AAGGAGGAGATCCGGCAGAG	GACGACCTTGAGCTGCTTCC
MH02g0466100	ACCATGCTGTCTTCGAGCAA	ACTGCATCCGTGACTTCCTG
MH02g0466300	CACGGTGACTGGTGTTGAGA	GTCACATCGGTAGTCCGCAT
MH02t0466400	ACGTTCCCGTAGCAGCATAG	CTGTCCCTGTTGCCCTGAAA
MH02t0466500	CTCTCGTTGGTGCTCGTCTTG	CTACTTGTCCTTCTTGCGCTTCT
MH02t0466600	CCGGCAAGAAATTCACCAAC	AGCGGCCAATGATCTCAGTC
MH02t0466700	GTCATCTTCGTCTTCCCCAGCC	TGGTTCCGGAGGTTTCGCTT
MH02t0466800	CCTAACTCGCCACCAAACCT	CCGATGGCCGGGATAGAAAT
MH02t0466900	TACATGAAGAACGGCCTCAT	GAGATGGTTACTGTTGCAGC
UBI	TGAAGACCCTGACTGGGAAG	CCGCCACGGAGCCTGAGGA

Table S4 Primers used for PCR amplify the *MH02t0466800* gene sequence.

**Genes**	**F**	**R**
Whole gene	ATGGCGATCGCGTCCATGGC	TTAGAGATGGTTACTGTTGCAGCTT
Anterior gene sequence	CAGTCTCTCGCGGTCTCTCG	GCGAGCAGCCAGAAGAACCA
Posterior gene sequence	CTTCTGCATCAGCTTCATCC	CCAATTGTAACAGATGGACCAA

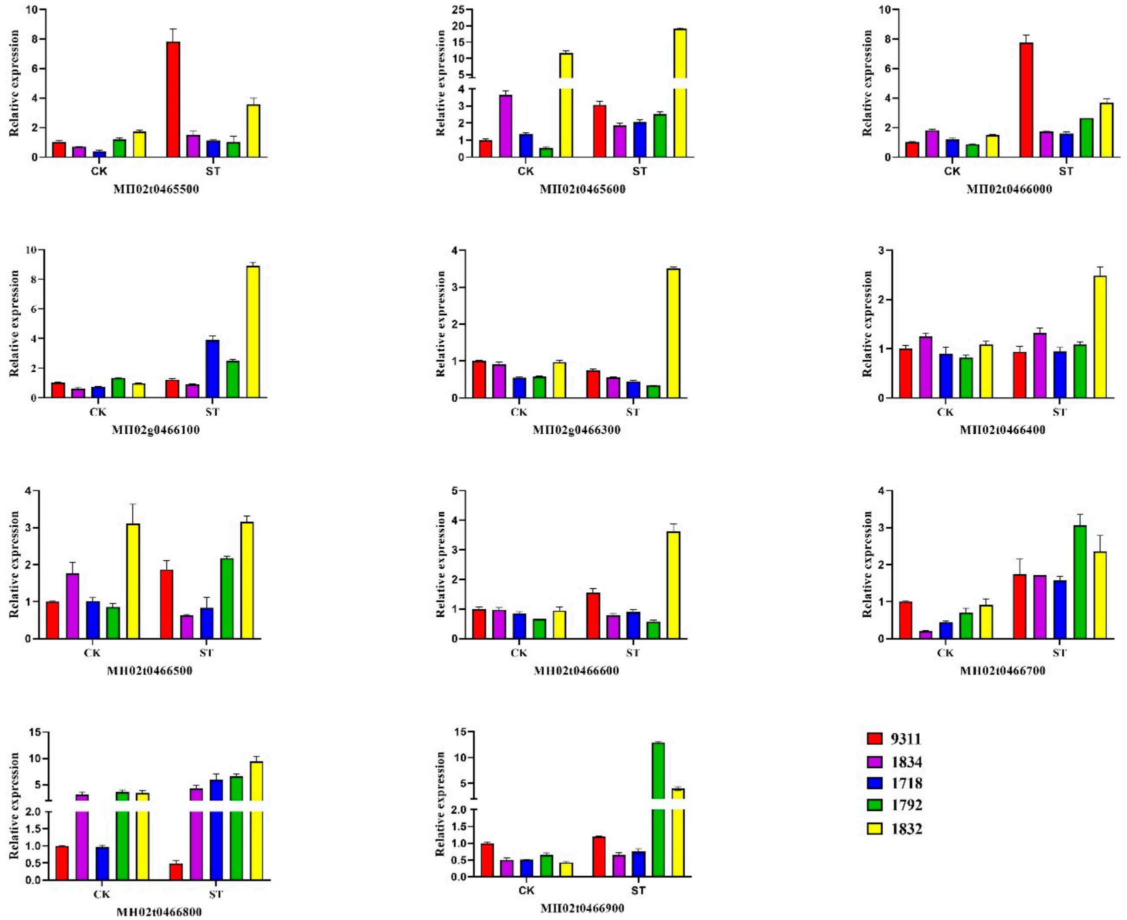

Figure S1. qPCR analysis of the candidate genes.

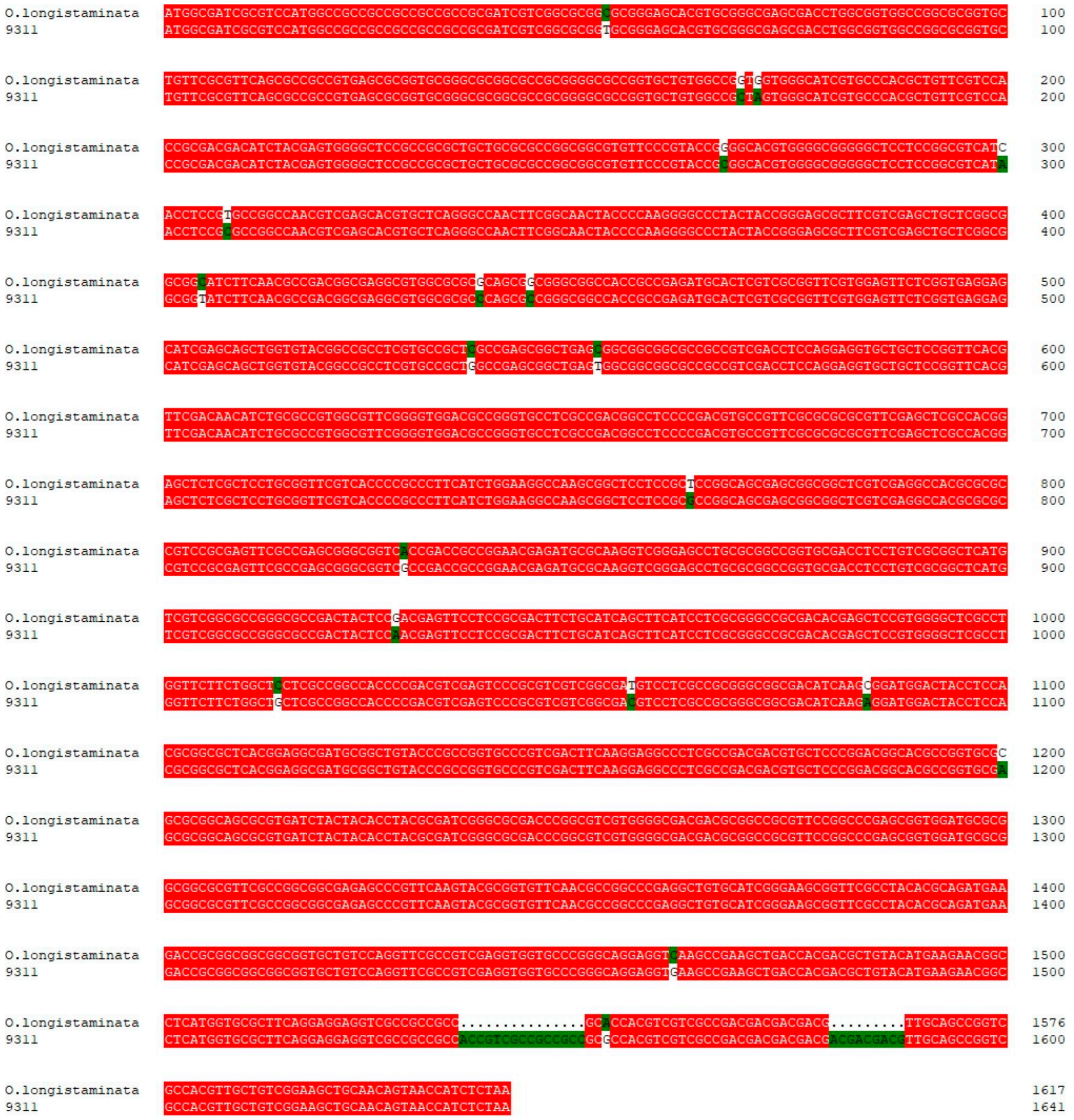

Figure S2. Sequence alignment of the candidate gene of *MH02t0466800* between *O. longistaminata* and 9311.

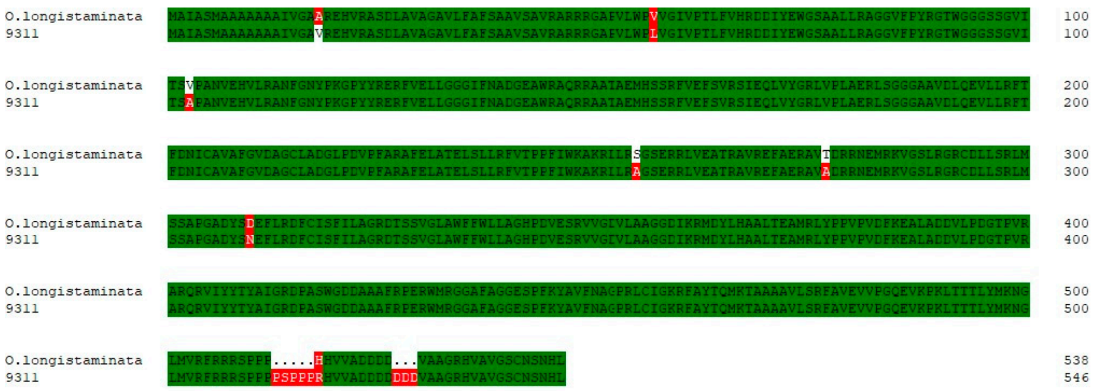

Figure S3. Amino acid sequence alignment of the candidate gene of *MH02t0466800* between *O. longistaminata* and 9311.

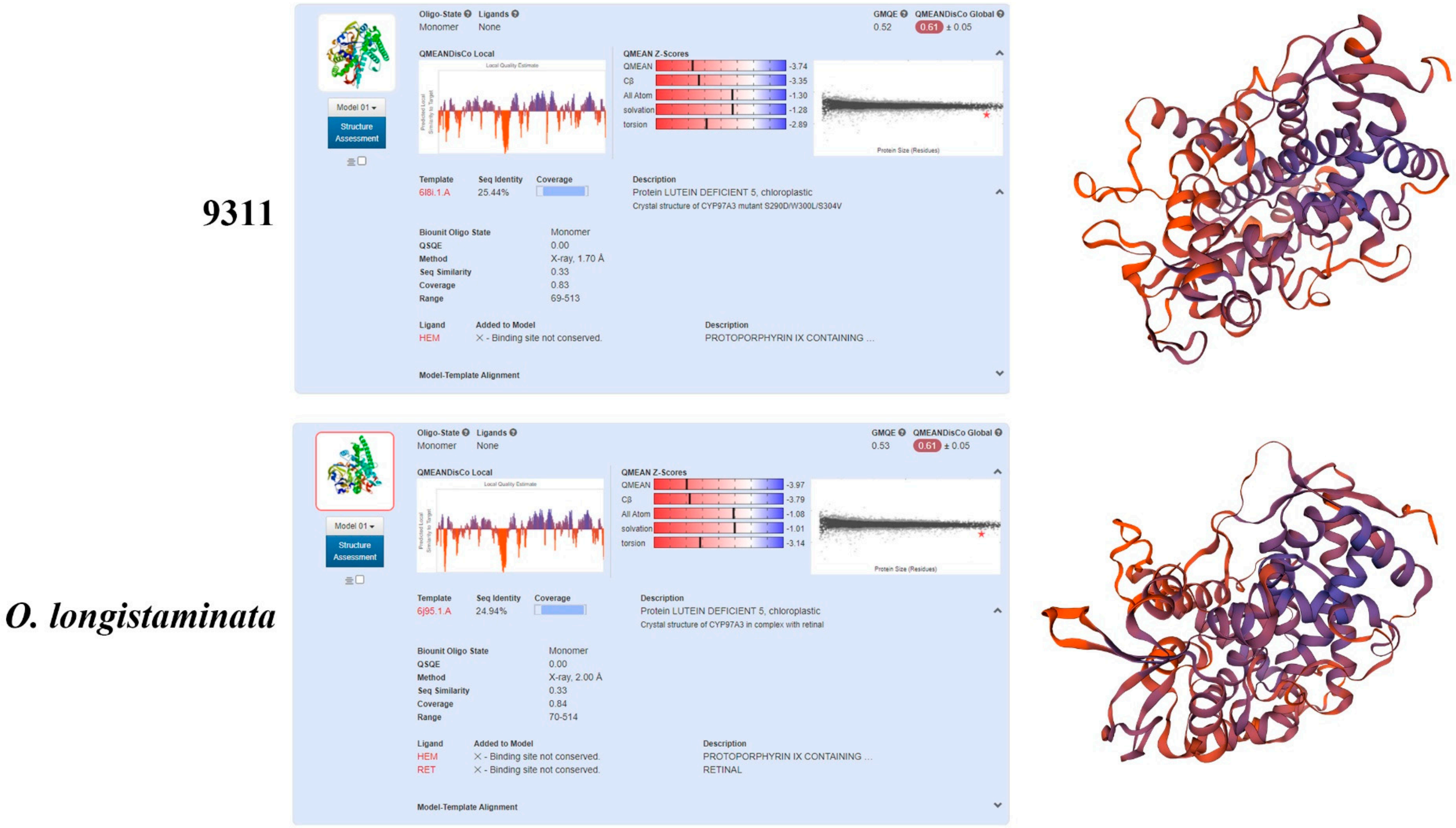

Figure S4. Predicted protein structure of *MH02t0466800* in *O. longistaminata* and 9311.
